# Clinical, epidemiological, and molecular characteristics of SARS-CoV-2 Infections among healthcare workers at a research center in the amazon region of BRAZIL from 2020 to 2022

**DOI:** 10.1007/s42770-024-01557-x

**Published:** 2024-11-01

**Authors:** Darciane Coelho Cordovil, Delana Andreza Melo Bezerra, Rayssa Layna Silva Bedran, Edvaldo Tavares Penha Junior, Dielle Monteiro Teixeira, Patricia Santos Lobo, Jones Anderson Monteiro Siqueira, Adinaura Gama Ramos, Amanda Mendes Silva, Kenny Costa Pinheiro, Jedson Cardoso Ferreira, Wanderley Dias Chagas Junior, Luana Soares Barbagelata, Fernando Neto Tavares, Mirleide Cordeiro Santos, Luana Silva Soares

**Affiliations:** 1 Seção de Ensino e Informação Científica, Programa de Pós-Graduação Em Virologia, Instituto Evandro Chagas, Ananindeua, Pará Brasil; 2 Seção de Virologia, Instituto Evandro Chagas, Secretaria de Vigilância em Saúde e Ambiente, Ministério da Saúde, Rodovia BR 316–KM 07, S/N, Levilândia, 67.030-000 Ananindeua, Pará Brasil

**Keywords:** COVID-19, Healthcare worker, SARS-CoV-2 variant

## Abstract

The coronavirus disease-2019 (COVID-19) pandemic has affected different sectors of society, and healthcare workers have been particularly impacted. This study aimed to describe the clinical, epidemiological, and molecular characteristics of SARS-CoV-2 infections among healthcare workers in Evandro Chagas Institute, a research reference center in Brazil, from October 2020 to July 2022. 845 samples were collected from individuals who presented clinical symptoms of respiratory infection. Nasopharyngeal positive samples were submitted through genome sequencing. Clinical, epidemiological, and the SARS-CoV-2 lineages (or variants) were analyzed. SARS-CoV-2 positivity was detected in 31.8% (269/845) of samples with a higher prevalence of females (60.2%). The highest SARS-CoV-2 positivity rates were reported in March 2021 (39%), January 2022 (65%), and July 2022 (56%). On clinical symptoms, arthralgia, chills, and diarrhea were statistically significantly detected in 2020; fever, runny nose, and arthralgia in 2021; runny nose, and cough in 2022. On molecular analysis of SARS-CoV-2, 66 samples (25.3%, 66/269) were sequenced and the most prevalent lineage was the Omicron, representing 57.6%. Studies on the epidemiological and clinical characteristics of HCW are essential to propose control measures and work management since research centers play a major role in surveillance to identify and monitor infectious diseases.

**Supplementary Information:**

The online version contains supplementary material available at 10.1007/s42770-024-01557-x.

In December 2019, the first human case of severe acute respiratory syndrome coronavirus 2 (SARS-CoV-2) was reported in Wuhan City of China’s Hubei Province and has rapidly spread globally [1]. The World Health Organization (WHO) subsequently declared it a global pandemic in March 2020 [2].

Since the outbreak of COVID-19, more than 700 million cases have been reported worldwide and caused more than 7 million deaths [3]. In Brazil, the first case was confirmed on February 26, 2020, and accumulated data indicate 38,883,865 confirmed cases and 713,026 deaths until September 2024 [4].

The disease was characterized as highly infectious, with the main clinical symptoms being fever, dry cough, fatigue, myalgia, and dyspnea, with cases evolving into acute respiratory distress syndrome, as well as septic shock, difficult-to-treat metabolic acidosis and hemorrhagic and coagulation dysfunction [5].

With the rapid spread of the disease, molecular analysis of SARS-CoV-2 demonstrated that mutations in the Spike protein change several viral characteristics, such as transmissibility, disease severity, drug resistance, and antigenicity. The emergence of new strains in several countries has raised additional public health concerns [6].

During pandemics it is common for health care workers (HCW), scientists, and managers to focus predominantly on the pathogen and the biological risk to understand the pathophysiological mechanisms involved and propose measures for preventing, containing, and treating the disease and health professionals are one of the most exposed categories at risk of infection [7, 8].

In this regard, the need to maintain essential services, such as public health laboratories, the advancement of research, and a rapid response to the health system, caused by the pandemic dynamic, had to rely on HCW involved directly or indirectly in confronting the pandemic. Preventing professional infections is necessary for maintaining health system’s capacity and reducing secondary transmission [9, 10].

It is important to know about SARS-CoV-2 infection in the workplace, to understand the epidemiology, and to recommend specific measures to ensure the protection of these professionals in this environment [10]. Therefore, this study aimed to describe clinical, epidemiological, and molecular characteristics of SARS-CoV-2 infections among HCW at Evandro Chagas Institute, a research reference center in the Amazon region of Brazil. This center plays a major role in surveillance and research in Brazil, as a center of excellence for identifying and monitoring infectious diseases.

## Methods

### Study population and sample collection

This study was conducted from October 2020 to July 2022 at the Evandro Chagas Institute, located in the metropolitan region of Belém-Pará. In April 2020, given the need for internal control to contain the COVID-19 pandemic, it was implemented preventive measures among HCW and the institution's internal public (researchers, students, outsourced professionals). The total population of HCW in institution is about 700 professionals. The measures adopted included testing for SARS-CoV-2 and the withdrawal of professionals diagnosed with COVID-19. Individuals who presented clinical symptoms of respiratory infection required testing for SARS-CoV-2 by submitting a standard questionnaire filled out with epidemiological and clinical information and subsequently collecting a nasopharyngeal swab.

### Extraction of viral RNA and SARS-CoV-2 detection

A total of 140 µL of samples were collected with combined swabs; then, viral RNA was extracted using QIAamp^®^ Viral RNA Mini Kits (QIAGEN, Germany) according to the manufacturer’s instructions. The isolated nucleic acid was subjected to the RT-qPCR method using primers and probes specific, according to IBMP Biomol One Step COVID-19 kit (IBMP, Brazil).

### Complete genome sequencing of SARS-CoV-2

Complete genome sequencing was performed using Illumina MiSeq platform using COVIDSEQ Kit (Illumina, San Diego, CA, USA). The data was assessed for quality using the FastQC software [11] and then the Trimmomatic [12] program was used to remove ends and reads with a quality of less than Phred 20 and reads with a size of less than 50 bp and possible adapters. The parameters used in Trimmomatic were Leading:3 Trailing:3 SLIDINGWINDOW 4:10 Minlen:50. The reads were aligned to the SARS-CoV-2 reference genome (NC_045512.2) using the BWA software [13]. The BWA-MEM algorithm was used in alignment and configured to run using default parameters. After alignment, files are generated in Binary Alignment Map (BAM) format. It is possible to obtain the final consensus sequences that can be further analyzed through manual curation using the Geneious software (v.9.1.8). The minimum coverage to produce the consensus sequences was 30x. The next step is to assign a lineage to each curated sequence using the PANGOLIN software (v.4.3.1) [14]. Finally, to validate the quality of the assembled and curated sequences, the NexClade web tool was used, available at 'https://clades.nextstrain.org/', which also provides information for assigning clades, calling mutations, and checking the quality of the sequences [15].

### Statistical analysis

Comparisons of COVID-19 infection rates in distinct groups were performed by the Bioestat v.5.3 program using the Chi-square test, simple logistic regression, Person's linear correlation, and G-Test, according to the sample size, with statistical inference with a significance level of 5% (*p* < 0.05).

## Results

**Fig. 1:**
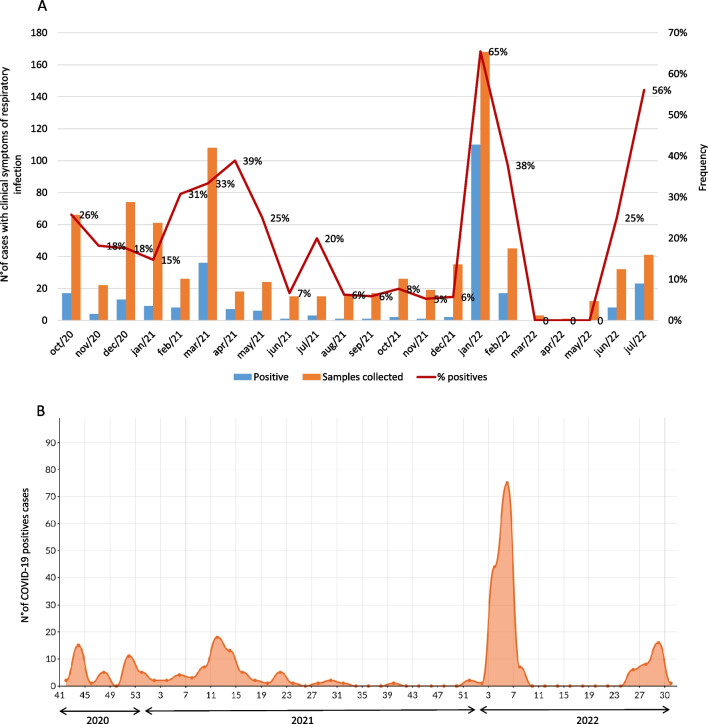
Positive for SARS-CoV-2 cases distribution among healthcare workers at a research reference center in Brazil, during October 2020 to July 2022. (**A**) Monthly and (**B**) Epidemiological weeks distribution

A total of 845 HCW were enrolled in the present study, 66.2% (559/845) were female, and 33.8% (286/845) were male. The overall prevalence rate of SARS-CoV-2 was 31.8% (269/845). Figure 1(A) shows the monthly frequencies of COVID-19 cases from October 2020 to July 2022. According to each year, SARS-CoV-2 was detected as follows: 20.8% (34/163) in 2020, 20.2% (77/380) in 2021 and 52.3% (158/302) in 2022. The highest positivity periods were from March 2021 (39%), January/2022 (65%) and July/2022 (56%). From March to May/2022, SARS-CoV-2 was not detected.

The distribution of cases according to epidemiological weeks (EW) is summarized in Fig. 1(B). The year-to-year prevalence was observed in the following periods: in 2020, the 43^rd^ week (October, 18 to 24) contributed with a peak of 15 positive cases; in 2021, there was a peak during the 11^th^ (18 cases) and 13^th^ (13 cases) weeks (March, 14 to April, 3); in 2022, the data collected showed a peak during 3^rd^ and 5^th^ epidemiological week with a total of 119 positive cases (January, 16 to February, 5).

Table 1 provides epidemiological aspects of COVID-19 infection. According to gender, females were more affected (60.2%) than males (39.8%) (*p*= 0.0159). No correlation was shown between age group and the risk of SARS-CoV-2 infection; however, the most affected group ranged between 20 to 39 years (48.3%), followed by 40 to 59 years (39.8%) (Table 1).

**Table 1:** General characteristics based on SARS-CoV-2 infection among healthcare workers at a research reference center in Brazil, from October 2020 to July 2022

Characteristics	SARS-CoV-2 infection						*p-value*
	Positive		Negative		Total		
	N	%	N	%	N	%	
Gender							
Female	162	60,2	397	68,9	559	66,2	
Male	107	39,8	179	31,1	286	33,8	*0.01
Age group (years)							
≤ 20	4	1,48	5	0,9	9	1,0	**0,65
20 to 39	130	48,3	285	49,5	415	49,1	**0,75
40 to 59	107	39,8	239	51,5	346	41,0	**0,64
≥ 60	28	10,4	47	8,1	75	8,9	**0,36
Total	269		576		845		

*Chi-square test, ** G test

Regarding clinical symptoms, it was reported that fever (*p-value* 0.0002 in 2021), runny nose (*p-value* 0. 005 in 2021 and *p-value* 0.0001 in 2022), arthralgia (*p-value* 0.02, *p-value* 0.03 in 2020 and 2021, respectively), cough (*p-value* 0.0001 in 2022), chills (*p-value* 0.002*; p-value* 0.01; *p-value* 0.0001 in 2020, 2021 and 2022 respectively), and diarrhea (*p-value* 0.02 in 2020) were statistically significant. Other symptoms were reported, such as nasal obstruction, myalgia, and headache (Table 2).

**Table 2:** Clinical characteristics of COVID-19 cases among healthcare workers at a research reference center in Brazil, from October 2020 to July 2022

Clinical symptoms	SARS-CoV-2 infection						
	Positive		Negative		Total		*Odds ratio(OD); p- value*
	N	%	N	%	N	%	
Fever							
No	143	26.7	393	73.3	536	63.4	2020(*OR*:3.1246;*p*:0,06); 2021 (*OR*:2.6070;*p*:0.002);
Yes	126	40.8	183	59.2	309	36.6	2022 (*OR*:1.4094; *p*:0.14)
Runny Nose							
No	96	27.6	252	72.4	348	41.2	2020(*OR*:1.1690;p:0.68); 2021(*OR*:0.4857;*p*:0.05);
Yes Nasal	173	34.8	324	65.2	497	58.8	2022 (*OR*:2.7986; p:0.0001)
obstruction							
No	181	30.4	415	69.3	596	70.5	2020(*OR*:0.5037;*p:*0.23); 2021(*OR*:1.2018;*p*:0.48);
Yes	89	35.7	160	64.3	249	29.5	2022(*OR*:1.7882; *p*:0.06)
Arthralgia							
No	248	31.1	549	68.9	797	94.3	2020(*OR*:4.2759;*p*:0.02); 2021(*OR*:2.7143;*p:*0.03);
Yes	22	45.8	26	54.2	48	5.7	2022 (*OR*:0.0396; *p*:089)
Myalgia							
No	201	31.5	437	68.5	638	75.5	2020(*OR*:2.2042;*p*:0.06); 2021(*OR*:0.8000;*p:*0.45);
Yes	69	33.3	138	66.7	207	24.5	2022 (*OR*:1.1916;*p*:0.51)
Cough							
No	65	20.4	253	79.6	318	37.6	2020(*OR*:2.1529;*p*:0.06); 2021(*OR*:1.6667;*p:*0.05);
Yes	205	38.9	322	61.1	527	62.4	2022 (*OR*:2.8085; *p*:0.0001)
Headache							
No	144	32.3	302	67.7	446	52.7	2020(*OR*:0.6682;*p*:0.30); 2021(*OR*:0.8435;*p:*0.50);
Yes	126	31.6	273	68.4	399	47.3	2022 (*OR*:1.2911; *p*:0.26)
Sore throat							
No	87	30.1	202	69.9	289	34.2	2020(*OR*:0.9190;*p*:0.82); 2021(*OR*:1.2868;*p:*0.38);
Yes	183	33.0	373	67.0	556	65.8	2022(*OR*:1.2460, *p*:0.41)
Chills							
No	203	28.0	522	72.0	725	85.8	2020(*OR*:9.000;*p*:0.002); 2021(*OR*:2.1750;*p:*0.01);
Yes	67	55.8	53	44.2	120	14.2	2022 (*OR*:3.6056; *p*:0.0001)
Dyspneia							
No	251	32.0	534	68.0	785	92.9	2020(*OR*:0.4583;*p*:0.46); 2021(*OR*:1.6182;*p:*0.19);
Yes	19	31.7	41	68.3	60	7.1	2022 (*OR*:1.9211; *p*:0.34)
Diarrhea							
No	229	32.9	468	67.1	697	82.5	2020(*OR*:2.6065;*p*:0.02); 2021(*OR*:0.8182;*p:*0.56);
Yes	41	27.7	107	72.3	148	17.5	2022(*OR*:1.1486; *p*:0.70)

^*^Logistic regression test

Concerning comorbidities and risk factors, of the 269 positive COVID-19 patients, 75 (27.8%) reported previous disease or risk factor: cardiovascular disease was the most frequent previous disease (41.8%), followed by diabetes (14.9%), asthma (17.9%), chronic pneumopathies (6%), chronic hematological disease (2.9%) and immunosuppression (2.9%). Among the risk factors related to COVID-19 were obesity (8%), pregnancy (6.7%), and smoking (5.3%). Nevertheless, no statistical significance was shown about comorbidities and SARS-CoV-2 infection (2020: *p-value* = 0.64; OD = 0.81; 2021: *p-value* = 0.78; OD: 0.91; 2022: *p-value* = 0.81; OD: 1.06).

**Fig. 2:**
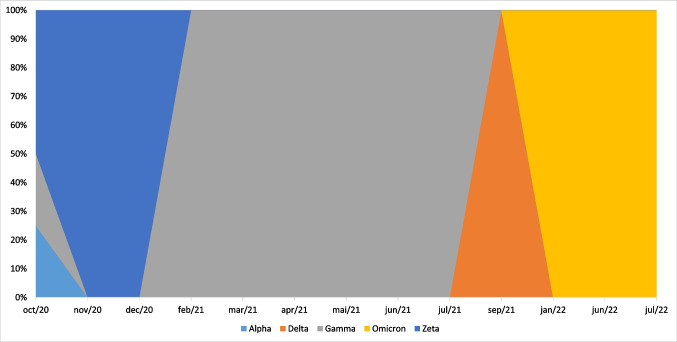
SARS-CoV-2 strains distribution over time according to the nomenclature proposed by the WHO Technical Advisory

On molecular analysis of SARS-CoV-2, 66 samples (25.3%, 66/269) were complete genome sequenced during the overall research period. The distribution of these specimens over time was: 10 (29.4%, 10/34) collected in 2020, 18 (23.3%, 18/77) from 2021, and 38 samples (24%, 38/158) from 2022. The most prevalent lineage was the Omicron lineage, representing 57.6% of the total cases, followed by Gamma (27.3%), Zeta (12.1%), Alpha (1.5%), and Delta (1.5%) lineages. The following variants were identified according to the periods. In 2020, the Alpha (B.1.1), Gamma (B.1.1.28) and Zeta (P.2) variants were detected in 10% (1), 10% (1) and 80% (8) of strains, respectively. In 2021, the Gamma (P.1, P.1.7) and Delta (AY.6) variants were found in 94.4% (17) and 5.6% (1) of the specimens, respectively. Between January and July 2022, the Omicron was predominantly found with a diversity of variants in 100% (38) of the strains (Fig. 2).

## Discussion

This cross-sectional study characterizes the SARS-CoV-2 infection profile in HCW at a research center in the Amazon region from Brazil, which is one of the most exposed categories at risk of infection during pandemics [7, 8]. In the present study, SARS-CoV-2 was detected in 31.8% of patients, a higher frequency when compared with studies conducted with HCW from Brazil (19.12%; 12.7%; 23.6%) [16–18], Egypt (6%) [19] and Italy (20.2%) [20]. Data from SARS-CoV-2 frequency depends on the population analyzed and diagnostic methods used [8, 16].

On temporal COVID-19 cases distribution, in 2020 the highest prevalence was during EW 43, a similar was observed in the northern region of Brazil where it represented a total of 12.7% (684,952) of national COVID-19 cases, being Pará state the second setting with the highest frequency of novel cases. This may be associated to Cirio de Nazare procession, a regional religious festivity in October in Pará state, where the research center is located. It is worth mentioning that the procession was canceled in 2020, even though the population kept the celebration among their families and friends, which could influence the transmission of SARS-CoV-2.

In 2022, EW 3 to 5 showed the highest prevalence of COVID-19 (111 cases), a period that recorded an increase of 40% in cases. By early 2022, the Omicron variant and its subvariants were becoming predominant in several regions. Omicron demonstrated an increased transmission capacity compared to previous variants, which may have contributed to the increase in cases [4]. The COVID-19 distribution of the present study followed the national scenario, and it was not possible to attribute the work environment as a site of infection, despite the possibility of exposure.

Regarding gender and age aspects, in the present study, a higher percentage of COVID-19 cases infected women (60.2%) and individuals aged 29–59 years (88.1%). In agreement with a study on the seroprevalence of COVID-19 cases in HCW in Ribeirão Preto, São Paulo, where 73.5% of women tested positive for COVID-19 [17]. Similar data were reported in Maringá, Paraná, where 59137 COVID-19 cases were analyzed and the most affected age group was between 20 and 59 years (76.19%) [21]. Our data reinforce that these age groups encompass the social and professional groups most subject to exposure and spread of the virus. Even as that mostly of HCW are women and consequently more exposed to infection.

With respect to clinical characteristics, in 2020, patients infected with SARS-CoV-2 were 2.6 times more susceptible to present diarrhea. Studies have shown that the gastrointestinal tract may also represent target organs for SARS-CoV-2 since it has pathogenic mechanisms similar to other coronaviruses, such as MERS-CoV and SARS-CoV, and based on evidence that ACE-2, the main receptor of SARS-CoV-2 is significantly expressed in the gastrointestinal tract and that this virus has acquired adaptation over time [22, 23]. Then, clinical data showed a correlation with previous information reported, suggesting that the virus creates mechanisms over time to spread the infection.

In 2021 it was observed that patients infected with SARS-COV-2 were about 2.6 times more likely to have a fever than uninfected patients, corroborating the study conducted in Rio Grande do Sul, where it was shown that individuals positive for SARS-CoV-2 had 4.2 times more fever than negative patients [24].

In 2022, COVID-19 cases demonstrated 2.7 times (OD; 2.7986) higher chance of presenting runny nose and 2.8 time (OD; 2.8085) cough. An epidemiological study carried out in December 2021 in Norway, identified runny nose/nasal obstruction as one of the main symptoms reported by 73% of positive cases [25]. In another study of participants who reported test results and symptoms on the ZOE COVID app, living in the United Kingdom, among those who tested positive when infected with the Omicron variant, the runny nose was also the most reported symptom, cited by 76.5% of participants, as well as persistent cough reported in 49.8% of cases [26]. It is worth mentioning that Omicron was reported in all sequenced specimens from 2022 and observed a modification in the COVID-19 symptomatology feature, suggesting changes in the behavior of SARS-CoV-2 infection and/or combined with the effectiveness of the vaccines offered to the population, attenuating the symptoms of the disease [27].

Regarding the comorbidities, the most prevalent were cardiovascular disease (41.8%) and diabetes (14.9%). Martono et al. (2023) [28] in a literature review related cardiovascular disease, diabetes, hypertension, and smoking as factors that may be associated with increased severity of COVID-19. In the present study, it was not possible to observe an association between risk factors and the worsening of infection, since one of the limitations was the lack of follow-up of the cohort to verify the outcome of the disease, however, it is important to mention that none of participating in the study died.

The present study related the same profile of SARS-CoV-2 variants circulation in Brazil, with B.1.1.28 and B.1.1.33 the most prevalent strains until October 2020 [29]. In the Amazon region, the B.1.1.28 strain circulated predominantly from May to December 2020, and replacement took place by P.1 lineage [30]. However, in the present study, the Zeta variant (P.2) was the most frequent in 2020, being found in 80% of samples. Zeta was first identified in October 2020 in Rio de Janeiro state, however, afterwards it was found to have been circulating in the country since July 2020, then, four months before its identification [31].

Gamma (P1) variant was detected in 94.4% of specimens in 2021, in agreement with other studies that demonstrated the emergence of this VOC from November 2020 in the Amazonas state (Northern Brazil), with a broad number of mutations, promoting a rapid spread to other Brazilian states, being associated with the second wave of COVID-19 collapsing the public health system in early 2021 [6, 30, 32].

The Omicron variant was reported in 100% of specimens from 2022, with a wide diversity of sub-variants. Similar data were reported from HCW across Lebanon between December 2021 and January 2022, where Omicron variant was the predominant VOC (90.6%) [33]. Andreis et al. (2023) [34] also described a substantial diversity of Omicron lineage among HCW and inpatients in southern, Brazil, during November 2022—January 2023. The accumulation of mutations in Spike protein over time boosted its rapid spread. Unlike the emergence of the Delta variant in mid-2021, which gradually replaced the Gamma lineage without an increase in SARS-CoV-2 cases, the Omicron variant resulted in a rapid replacement of Delta lineages, with exponential growth in COVID-19 cases [35].

This study had some limitations: as it was a cross-sectional study, it was not possible to verify the outcome of the case; it was not possible to include asymptomatic patients and reinfection cases; there was no testing for other respiratory agents. Our findings support previous literature demonstrating the COVID-19 distribution profile is like that observed in the general population, despite limited studies describing lineages among HCW in Brazil. However, studies on the epidemiological and clinical characteristics of HCW are essential to propose control measures and work management to break the transmission of disease at laboral places, since research centers play a major role in surveillance to identify and monitor infectious diseases.

## Supplementary Information

Below is the link to the electronic supplementary material.

Supplementary file1 Information about SARS-CoV-2 sequences was generated in this study (PDF 54 KB)

## Data Availability

The datasets presented in this study can be found in online repositories. The names of the repository/repositories and accession number(s) can be found in the article/Supplementary Material.
