# Supplementary material for: Clinical, epidemiological, and molecular characteristics of SARS-CoV-2 Infections among healthcare workers at a research center in the amazon region of BRAZIL from 2020 to 2022
Source: Braz J Microbiol. 2024 Nov 1;56(1):529–36. doi: 10.1007/s42770-024-01557-x (PMC11885708; doi:10.1007/s42770-024-01557-x)
Supplement: Supplementary file 1 — Supplementary file1 Information about SARS-CoV-2 sequences was generated in this study (PDF 54 KB) [file 42770_2024_1557_MOESM1_ESM.pdf]

Supplementary Material table 1

Virus name	Accession ID Gisaid	Collection date	Submission date to GISAID	Pango Version	Nextclade Version	Pangolin classification	SARS-CoV-2 variant
hCoV-19/Brazil/PA-IEC-176408/2020	EPI_ISL_16893918	2020-10-02	2023-02-10	V.4.3.1	Nextclade v2.11.0	B.1.1	Alpha
hCoV-19/Brazil/PA-IEC-176485/2020	EPI_ISL_16893919	2020-10-15	2023-02-10	V.4.3.1	Nextclade v2.11.0	B.1.1.28	Gamma
hCoV-19/Brazil/PA-IEC-176597/2020	EPI_ISL_16893920	2020-10-21	2023-02-10	V.4.3.1	Nextclade v2.11.0	P.2	Zeta
hCoV-19/Brazil/PA-IEC-176734/2020	EPI_ISL_16893921	2020-10-29	2023-02-10	V.4.3.1	Nextclade v2.11.0	P.2	Zeta
hCoV-19/Brazil/PA-IEC-176761/2020	EPI_ISL_16893922	2020-11-12	2023-02-10	V.4.3.1	Nextclade v2.11.0	P.2	Zeta
hCoV-19/Brazil/PA-IEC-176778/2020	EPI_ISL_16893923	2020-11-19	2023-02-10	V.4.3.1	Nextclade v2.11.0	P.2	Zeta
hCoV-19/Brazil/PA-IEC-176830/2020	EPI_ISL_16893924	2020-12-03	2023-02-10	V.4.3.1	Nextclade v2.11.0	P.2	Zeta
hCoV-19/Brazil/PA-IEC-176906/2020	EPI_ISL_16893925	2020-12-09	2023-02-10	V.4.3.1	Nextclade v2.11.0	P.2	Zeta
hCoV-19/Brazil/PA-IEC-176944/2020	EPI_ISL_16893926	2020-12-14	2023-02-10	V.4.3.1	Nextclade v2.11.0	P.2	Zeta
hCoV-19/Brazil/PA-IEC-177012/2020	EPI_ISL_16893927	2020-12-21	2023-02-10	V.4.3.1	Nextclade v2.11.0	P.2	Zeta
hCoV-19/Brazil/PA-IEC-177628/2021	EPI_ISL_1164972	2021-02-02	2021-03-05	V.4.3.1	Nextclade v1.13.0	P.1	Gamma
hCoV-19/Brazil/PA-IEC-178310/2021	EPI_ISL_12140053	2021-03-04	2022-04-19	V.4.3.1	Nextclade v1.13.0	P.1	Gamma
hCoV-19/Brazil/PA-IEC-178387/2021	EPI_ISL_12140054	2021-03-04	2022-04-19	V.4.3.1	Nextclade v1.13.0	P.1	Gamma
hCoV-19/Brazil/PA-IEC-178394/2021	EPI_ISL_12140055	2021-03-10	2022-04-19	V.4.3.1	Nextclade v1.13.0	P.1	Gamma
hCoV-19/Brazil/PA-IEC-178398/2021	EPI_ISL_12140056	2021-03-11	2022-04-19	V.4.3.1	Nextclade v1.13.0	P.1	Gamma
hCoV-19/Brazil/PA-IEC-178483/2021	EPI_ISL_12140057	2021-03-16	2022-04-19	V.4.3.1	Nextclade v1.13.0	P.1	Gamma
hCoV-19/Brazil/PA-IEC-178493/2021	EPI_ISL_12140058	2021-03-17	2022-04-19	V.4.3.1	Nextclade v1.13.0	P.1	Gamma
hCoV-19/Brazil/PA-IEC-178502/2021	EPI_ISL_2488795	2021-03-17	2021-06-11	V.4.3.1	Nextclade v2.11.0	P.1	Gamma
hCoV-19/Brazil/PA-IEC-178515/2021	EPI_ISL_2488796	2021-03-22	2021-06-11	V.4.3.1	Nextclade v2.11.0	P.1	Gamma
hCoV-19/Brazil/PA-IEC-178527/2021	EPI_ISL_12140059	2021-03-23	2022-04-19	V.4.3.1	Nextclade v1.13.0	P.1	Gamma
hCoV-19/Brazil/PA-IEC-178544/2021	EPI_ISL_12140061	2021-03-29	2022-04-19	V.4.3.1	Nextclade v1.13.0	P.1	Gamma
hCoV-19/Brazil/PA-IEC-178587/2021	EPI_ISL_12140065	2021-03-31	2022-04-19	V.4.3.1	Nextclade v1.13.0	P.1	Gamma
hCoV-19/Brazil/PA-IEC-178680/2021	EPI_ISL_12140062	2021-04-07	2022-04-19	V.4.3.1	Nextclade v1.13.0	P.1	Gamma
hCoV-19/Brazil/PA-IEC-179068/2021	EPI_ISL_2488813	2021-05-17	2021-06-11	V.4.3.1	Nextclade v2.11.0	P.1	Gamma
hCoV-19/Brazil/PA-IEC-179309/2021	EPI_ISL_12140063	2021-06-09	2022-04-19	V.4.3.1	Nextclade v1.13.0	P.1	Gamma
hCoV-19/Brazil/PA-IEC-179823/2021	EPI_ISL_6721823	2021-07-19	2021-11-25	V.4.3.1	Nextclade v2.11.0	P.1.7	Gamma
hCoV-19/Brazil/PA-IEC-179825/2021	EPI_ISL_6721832	2021-07-19	2021-11-25	V.4.3.1	Nextclade v2.11.0	P.1	Gamma
hCoV-19/Brazil/PA-IEC-180387/2021	EPI_ISL_6721547	2021-09-20	2021-11-25	V.4.3.1	Nextclade v2.11.0	AY.6	Delta
hCoV-19/Brazil/PA-IEC-181415/2022	EPI_ISL_9411948	2022-01-12	2022-02-02	V.4.3.1	Nextclade v1.13.0	BA.1.14.1	Omicron
hCoV-19/Brazil/PA-IEC-181416/2022	EPI_ISL_9411949	2022-01-12	2022-02-02	V.4.3.1	Nextclade v1.13.0	BA.1.14.1	Omicron
hCoV-19/Brazil/PA-IEC-181419/2022	EPI_ISL_9411950	2022-01-12	2022-02-02	V.4.3.1	Nextclade v1.13.0	BA.1.14.1	Omicron
hCoV-19/Brazil/PA-IEC-181422/2022	EPI_ISL_9411951	2022-01-13	2022-02-02	V.4.3.1	Nextclade v1.13.0	BA.1.1.14	Omicron
hCoV-19/Brazil/PA-IEC-181551/2022	EPI_ISL_9411954	2022-01-17	2022-02-02	V.4.3.1	Nextclade v1.13.0	BA.1.1	Omicron
hCoV-19/Brazil/PA-IEC-181512/2022	EPI_ISL_9411952	2022-01-18	2022-02-02	V.4.3.1	Nextclade v1.13.0	BA.1.14.1	Omicron
hCoV-19/Brazil/PA-IEC-181513/2022	EPI_ISL_9411953	2022-01-18	2022-02-02	V.4.3.1	Nextclade v1.13.0	BA.1.14.1	Omicron
hCoV-19/Brazil/PA-IEC-181591/2022	EPI_ISL_9411956	2022-01-20	2022-02-02	V.4.3.1	Nextclade v1.13.0	BA.1.14.1	Omicron
hCoV-19/Brazil/PA-IEC-181610/2022	EPI_ISL_9411957	2022-01-21	2022-02-02	V.4.3.1	Nextclade v1.13.0	BA.1.1	Omicron
hCoV-19/Brazil/PA-IEC-181627/2022	EPI_ISL_9411959	2022-01-24	2022-02-02	V.4.3.1	Nextclade v1.13.0	BA.1.14.1	Omicron
hCoV-19/Brazil/PA-IEC-182725/2022	EPI_ISL_13726320	2022-06-13	2022-07-08	V.4.3.1	Nextclade v1.13.0	BA.5.2.1	Omicron
hCoV-19/Brazil/PA-IEC-182726/2022	EPI_ISL_13726321	2022-06-13	2022-07-08	V.4.3.1	Nextclade v1.13.0	BA.5.2.1	Omicron
hCoV-19/Brazil/PA-IEC-182752/2022	EPI_ISL_13726322	2022-06-14	2022-07-08	V.4.3.1	Nextclade v1.13.0	BA.5.2.1	Omicron
hCoV-19/Brazil/PA-IEC-182801/2022	EPI_ISL_13726323	2022-06-21	2022-07-08	V.4.3.1	Nextclade v1.13.0	BA.5.5	Omicron
hCoV-19/Brazil/PA-IEC-182802/2022	EPI_ISL_13726324	2022-06-21	2022-07-08	V.4.3.1	Nextclade v1.13.0	BA.5.2.1	Omicron
hCoV-19/Brazil/PA-IEC-182815/2022	EPI_ISL_16893928	2022-06-22	2023-02-10	V.4.3.1	Nextclade v1.13.0	BA.4.1	Omicron
hCoV-19/Brazil/PA-IEC-182914/2022	EPI_ISL_16893930	2022-06-28	2023-02-10	V.4.3.1	Nextclade v1.13.0	BA.5.1.15	Omicron
hCoV-19/Brazil/PA-IEC-182973/2022	EPI_ISL_16893931	2022-06-30	2023-02-10	V.4.3.1	Nextclade v1.13.0	BA.5.1.15	Omicron
hCoV-19/Brazil/PA-IEC-183170/2022	EPI_ISL_16893932	2022-07-06	2023-02-10	V.4.3.1	Nextclade v1.13.0	BA.5.2.1	Omicron
hCoV-19/Brazil/PA-IEC-183181/2022	EPI_ISL_16893933	2022-07-06	2023-02-10	V.4.3.1	Nextclade v1.13.0	BA.5.2.1	Omicron
hCoV-19/Brazil/PA-IEC-183182/2022	EPI_ISL_16893934	2022-07-07	2023-02-10	V.4.3.1	Nextclade v1.13.0	BA.5.2.1	Omicron
hCoV-19/Brazil/PA-IEC-183184/2022	EPI_ISL_16893935	2022-07-07	2023-02-10	V.4.3.1	Nextclade v1.13.0	BA.5.2.1	Omicron
hCoV-19/Brazil/PA-IEC-183185/2022	EPI_ISL_16893936	2022-07-07	2023-02-10	V.4.3.1	Nextclade v1.13.0	BF.39	Omicron
hCoV-19/Brazil/PA-IEC-183225/2022	EPI_ISL_16893937	2022-07-11	2023-02-10	V.4.3.1	Nextclade v1.13.0	BA.4	Omicron
hCoV-19/Brazil/PA-IEC-183226/2022	EPI_ISL_16893938	2022-07-12	2023-02-10	V.4.3.1	Nextclade v1.13.0	BA.5.1	Omicron
hCoV-19/Brazil/PA-IEC-183227/2022	EPI_ISL_16893939	2022-07-12	2023-02-10	V.4.3.1	Nextclade v1.13.0	BA.4.1	Omicron
hCoV-19/Brazil/PA-IEC-183273/2022	EPI_ISL_16893940	2022-07-13	2023-02-10	V.4.3.1	Nextclade v1.13.0	BA.5.1	Omicron
hCoV-19/Brazil/PA-IEC-183274/2022	EPI_ISL_16893941	2022-07-14	2023-02-10	V.4.3.1	Nextclade v1.13.0	BA.5.2.1	Omicron
hCoV-19/Brazil/PA-IEC-183277/2022	EPI_ISL_16893942	2022-07-14	2023-02-10	V.4.3.1	Nextclade v1.13.0	BA.5.2.1	Omicron
hCoV-19/Brazil/PA-IEC-183280/2022	EPI_ISL_16893943	2022-07-14	2023-02-10	V.4.3.1	Nextclade v1.13.0	BA.5.1	Omicron
hCoV-19/Brazil/PA-IEC-183281/2022	EPI_ISL_16893944	2022-07-14	2023-02-10	V.4.3.1	Nextclade v1.13.0	BA.5.2.1	Omicron
hCoV-19/Brazil/PA-IEC-183290/2022	EPI_ISL_16893945	2022-07-14	2023-02-10	V.4.3.1	Nextclade v1.13.0	BA.5.1	Omicron
hCoV-19/Brazil/PA-IEC-183361/2022	EPI_ISL_16893946	2022-07-19	2023-02-10	V.4.3.1	Nextclade v1.13.0	BA.5.1	Omicron
hCoV-19/Brazil/PA-IEC-183362/2022	EPI_ISL_16893947	2022-07-19	2023-02-10	V.4.3.1	Nextclade v1.13.0	BA.5.1.22	Omicron
hCoV-19/Brazil/PA-IEC-183380/2022	EPI_ISL_16893948	2022-07-19	2023-02-10	V.4.3.1	Nextclade v1.13.0	BA.4	Omicron
hCoV-19/Brazil/PA-IEC-183464/2022	EPI_ISL_16893949	2022-07-20	2023-02-10	V.4.3.1	Nextclade v1.13.0	BA.4	Omicron
hCoV-19/Brazil/PA-IEC-183465/2022	EPI_ISL_16893950	2022-07-21	2023-02-10	V.4.3.1	Nextclade v1.13.0	BA.5.2.1	Omicron
hCoV-19/Brazil/PA-IEC-183682/2022	EPI_ISL_16893952	2022-07-29	2023-02-10	V.4.3.1	Nextclade v1.13.0	BA.5.2.1	Omicron
